# U-shaped association of serum uric acid with all-cause mortality in patients with hyperlipidemia in the United States: a cohort study

**DOI:** 10.3389/fcvm.2023.1165338

**Published:** 2023-05-23

**Authors:** Lihua Huang, Zhanpeng Lu, Xiaoyan You, Chunsheng Zou, Liuliu He, Jingxiang Xie, Xiaoqing Zhou

**Affiliations:** ^1^ Department of Clinical Laboratory, The Second Affiliated Hospital of Gannan Medical University, Ganzhou, China; ^2^ Department of Critical Care, The Eighth Affiliated Hospital of Sun Yat sen University, Shenzhen, China; ^3^ General Surgery Department, The Second Affiliated Hospital of Gannan Medical University, Ganzhou, China

**Keywords:** uric acid, hyperlipidemia, NHANES, mortality, U-shaped

## Abstract

**Background:**

Serum uric acid (SUA) interferes with lipid metabolism and is considered an independent risk factor for atherosclerosis, a major complication in patients with hyperlipidemia. However, the effects of uric acid levels on mortality in hyperlipidemic patients has yet to be sufficiently determined. In this study, we aimed to assess the association between all-cause mortality and SUA in a hyperlipidemic population.

**Methods:**

To determine mortality rates, we obtained data for 20,038 hyperlipidemia patients from the U.S. National Health and Nutrition Examination Surveys (NHANES) 2001–2018 and National Death Index. To examine the all-cause mortality effect of SUA, multivariable Cox regression models, restricted cubic spline models, and two pairwise Cox regression models were used.

**Results:**

Over a median follow-up of 9.4 years, a total of 2079 deaths occurred. Mortality was examined according to SUA level quintiles: <4.2, 4.3–4.9, 5.0–5.7, 5.8–6.5, and >6.6 mg/dl. In multivariable analysis using 5.8–6.5 mg/dl SUA as a reference, the hazard ratios (95% confidence interval) of all-cause mortality across the five groups were 1.24 (1.06–1.45), 1.19 (1.03–1.38), 1.07 (0.94–1.23), 1.00 (reference), and 1.29 (1.13–1.48), respectively. According to a restricted cubic spline, we noted a U-shaped relationship between SUA and all-cause mortality. The inflection point was approximately 6.30 mg/dl, with hazard ratios of 0.91 (0.85–0.97) and 1.22 (1.10–1.35) to the left and right of the inflection point, respectively. In both sexes, SUA was characterized by a U-shaped association, with inflection points at 6.5 and 6.0 mg/dl for males and females, respectively.

**Conclusion:**

Using nationally representative NHANES data, we identified a U-shaped association between SUA and all-cause mortality in participants with hyperlipidemia.

## Introduction

Hyperlipidemia is a disease characterized by abnormally high blood levels of lipids or lipoproteins attributable to abnormalities in lipid metabolism or function associated with eating disorders, obesity, genetic disorders such as hereditary hypercholesterolemia, or other conditions such as diabetes (1). In the United States and Europe, more than 3 million adults are currently diagnosed with hyperlipidemia, and the number of individual with this condition continues to escalate (2). Hyperlipidemia substantially reduces patient quality of life and contributes to a two- to three-fold increase in the risk of cardiovascular disease (CVD) (3). The increase in associated mortality has highlighted the urgent need to develop novel strategies that can be adopted to prevent the disease, to identify new risk factors that can contribute to reducing disease severity, and to gain a sufficient understanding of the measures that can be taken to eliminate these risk factors.

In humans, uric acid is the final enzymatic product of purine nucleoside and free base degradation. Elevated levels of serum uric acid (SUA) are widely recognized as a risk factor for macrovascular and microvascular complications, and as a high-risk factor for CVD, hyperuricemia is particularly common in patients with hyperlipidemia. It has been established that SUA levels are proportional to triglyceride (TG), total cholesterol (TC), and low-density lipoprotein (LDL) levels (4), and the findings of an *in vitro* study have indicated that SUA promotes the oxidation of LDL, which is considered to be a key event in atherosclerotic plaque formation (5). The findings of several epidemiological studies have thus provided evidence to indicate a clear independent association between elevated SUA levels and an increased incidence of atherosclerotic disease or mortality. Interestingly, however, numerous experimental studies have demonstrated that uric acid has numerous benefits related to its antioxidant properties (6, 7). Indeed, uric acid circulating at high concentrations is considered one of the main antioxidants in plasma, which protects cells from oxidative damage, and can thereby contribute to prolonging human life span and reducing the risk of carcinogenesis (8).

Given the dual antioxidant and pro-oxidant properties of SUA, there is currently a lack of consensus regarding the association between SUA levels and adverse outcomes, and studies conducted to date have detected U-shaped (9), linear (10), and no (11) relationships between SUA concentrations and cardiovascular disease and total mortality risk in different populations. Given the complex biological activity of uric acid, the pros and cons of stringent hypo-uric acid therapy in hyperlipidemic populations, and the target values for lowering uric acid, need to be closely assessed. At present, however, evidence regarding an association between SUA levels and all-cause mortality in hyperlipidemia patients remains inconclusive. Accordingly, identifying such an association could provide new insights into the management of uric acid levels in patients with hyperlipidemia, particularly intensive anti-uric acid therapy in patients with hyperlipidemia combined with hyperuricemia. Toward this end, we sought in this study to assess the association between SUA levels and all-cause mortality in a large, nationally representative sample of US adults with hyperlipidemia.

## Materials and methods

**Figure 1:**
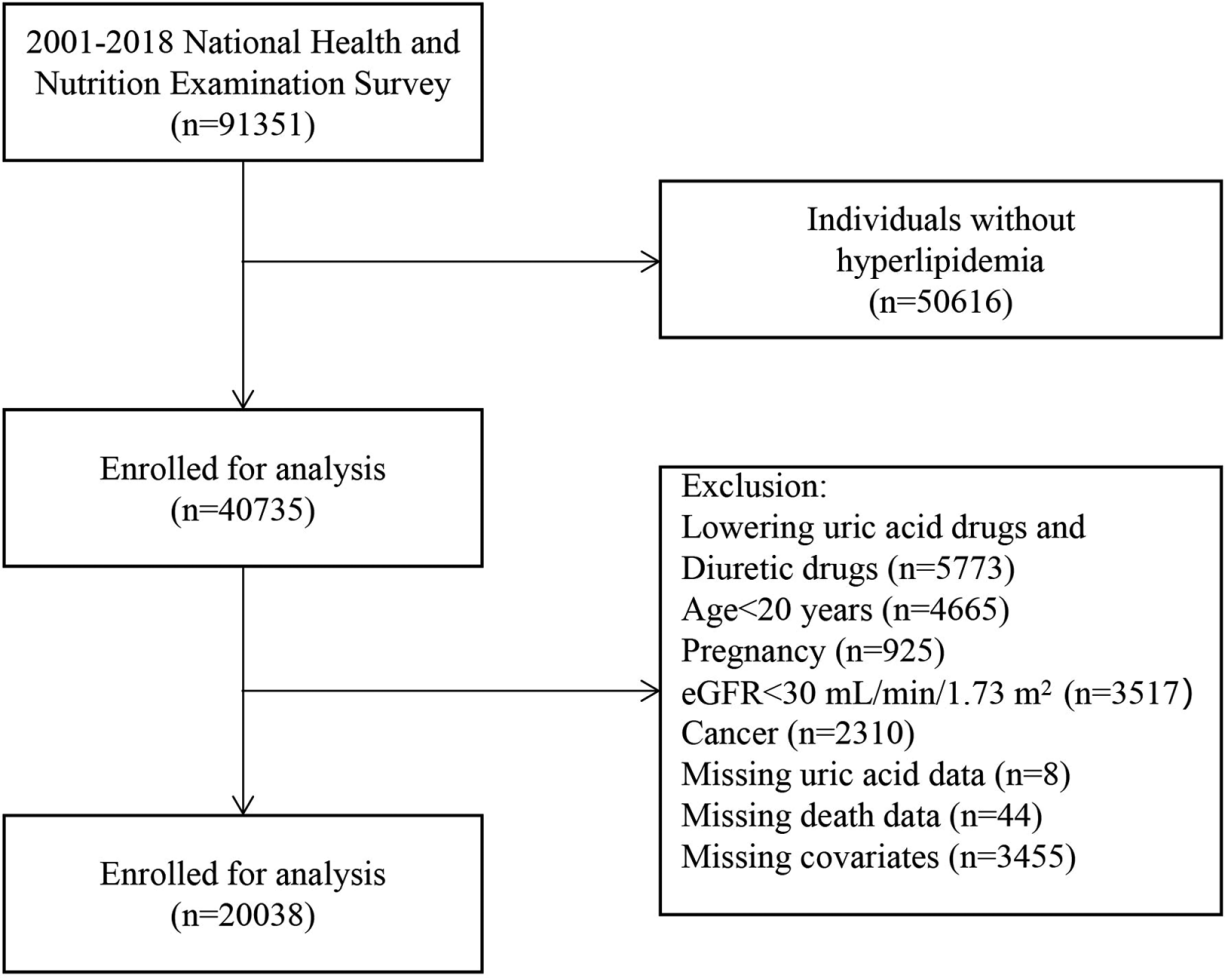
Study flowchart.

The National Health and Nutrition Examination Survey (NHANES) is conducted by the National Center for Health Statistics (NCHS), a division of the U.S. Centers for Disease Control and Prevention (CDC). The purpose of this national survey is to assess the health status of the entire population of the United States. Data are collected annually on a 2-year cycle using a multi-stage probability sampling method to generate population-level estimates. We calculated the number of non-institutionalized civilians using NHANES data obtained for the period between 2001 and 2018 and a design variable. In addition, we retrieved and combined demographic data, disease details, laboratory data, and questionnaire data on disease classification, which were measured at enrollment. A total of 40,735 subjects with hyperlipidemia were enrolled in the NHANES study from 2001 to 2018. To exclude the effects of certain baseline factors on SUA levels, we excluded patients based on the following criteria: pregnancy at baseline (*n* = 925), the use of uric acid-lowering drugs and diuretics (*n* = 5,773), an estimated glomerular filtration rate (eGFR) value <30 ml/min/1.73 m^2^ (*n* = 3,517), age <20 years (*n* = 4,665), having cancer at baseline (*n* = 2,310), missing SUA data (*n* = 8), missing death status (*n* = 44), and missing covariates (*n* = 3,455). Having excluding these individuals, the remaining 20,038 participants were included for analysis (Figure 1). The Institutional Review Board of the CDC National Center for Health Statistics has authorized NHANES to make the research data available to the public on its home page (http://www.cdc.gov/nchs/nhanes.html). No ethical approval was required for conducting this study.

### Serum uric acid measurement

SUA measurements were performed using a Beckman Synchron LX20 analyzer in the 2001–2007 NHANES, a Beckman UniCel DxC800 Synchron analyzer in the 2008–2016 NHANES, and a Roche Cobas 6,000 analyzer in the 2017–2018 NHANES. The analysis of SUA in combination with multiple cycles of NHANES has been reported previously (12). The study groups with hyperlipidemia were classified into five groups based on their SUA quintiles. The cut-off values for grouping were as follows: Quintile 1 (<4.2 mg/dl), Quintile 2 (4.3–4.9 mg/dl), Quintile 3 (5.0–5.7 mg/dl), Quintile 4 (5.8–6.5 mg/dl), and Quintile 5 (>6.6 mg/dl).

All laboratory variables were assessed using validated protocols and procedures. Details can be found at https://www.cdc.gov/nchs/nhanes.

### Definition of hyperlipidemia

Hyperlipidemia was defined as triglyceride (TG) levels ≥150 mg/dl (1.7 mmol/L) or total cholesterol (TC) ≥200 mg/dl (5.18 mmol/L), low-density lipoprotein (LDL) ≥130 mg/dl (3.37 mmol/L), or high-density lipoprotein (HDL) <40 mg/dl (1.04 mmol/L) in men and <50 mg/dl (1.30 mmol/L) in women (13). Additionally, participants who reported the use of cholesterol-lowering medications were also categorized as having hyperlipidemia.

### Covariates

For each of the enrolled participants, we analyzed age, gender, race/ethnicity, body mass index (BMI), education level, poverty income ratio, smoking status, diabetes, cardiovascular disease (CVD), hypertension, stroke, hypolipidemic medications, healthy eating index, physical activity, alcohol intake, estimated glomerular filtration rate (eGFR), TC, TG, high-density lipoprotein cholesterol (HDL-C), and low-density lipoprotein cholesterol (LDL-C). Age was classified at baseline and used in table classifications (<65 and ≥65) and as a continuous variable in regression analysis. Race/ethnicity was defined as Mexican American, other Hispanic, non-Hispanic white, non-Hispanic black, or other (including multi-racial). Education level was categorized into three levels: <9 years, 9–13 years, and ≥13 years. For the income-to-poverty ratio ($ income/threshold), the ratio of household income to poverty threshold was based on the number of persons in a household or unrelated individuals, and household income was divided by poverty guidelines based on the size of the household, the appropriate year, and the state in which it was calculated. On the basis of their responses to a series of questions, subjects were classified as current smokers, former smokers, or never a smoker. Current or former smokers, were those individuals who have smoked at least 100 cigarettes during his or her lifetime. Hypertension was defined as systolic and diastolic blood pressures ≥140 and ≥90 mm Hg, respectively, self-reported hypertension subsequently diagnosed by a physician, or elevated blood pressure requiring antihypertensive medication. Recorded systolic and diastolic blood pressures were the average of up to three blood pressure measurements. Diabetes was defined as being diagnosed by a physician as diabetic or taking insulin or glucose-lowering medication, or having a glycosylated hemoglobin level ≥6.5% or a fasting blood glucose level ≥126 mg/dl. CVD was defined as a self-reported diagnosis of congestive heart failure, angina, heart attack, or coronary heart disease by the participants (14). Data on a physician-diagnosed history of cancer, and stroke were self-reported. Hypolipidemic medication (within the past 30 days) was defined as no (no medication was used), other (use of medications other than lipid-lowering medications), and yes (use of lipid-lowering medications). In the category of lipid-lowering drugs, statins represented 89.6%, followed by gemfibrozil at 3.2%, fenofibrate at 2.5%, and other drugs at 4.7%. Healthy eating index values were calculated according to the HEI-2015 guidelines, with higher scores indicating a better quality of diet. The eGFR was calculated using the Chronic Kidney Disease Epidemiology Collaboration equation. In terms of physical activity, those participating in moderate or vigorous exercise, fitness programs, or recreational activities for more than 10 min per week were defined as physically active. Participants were considered inactive if they did not exercise for more than 10 min per week (15). The amount of alcohol consumed was determined on the basis of the 24-h dietary recall of participants. Current alcohol intake was categorized as none (0 g/day), moderate drinking (0.1–27.9 g/day for men and 0.1–13.9 g/day for women), or heavy drinking (≥28 g/day for men and ≥14 g/day for women) (12).

### Mortality

Using the National Mortality Index, we collected data on mortality status and follow-up information for all individuals as of December 31, 2019.

### Statistical analysis

Descriptive statistical analysis was performed for data obtained from all patients. Categorical variables are expressed as numbers and percentages. For normally distributed data, continuous variables are expressed as the means and standard deviation (SD) or the medians and interquartile range. For categorical, normally distributed, and non-normally distributed continuous variables, we used the *χ*^2^ test, one-way ANOVA, and the Kruskal–Wallis test, respectively. We conducted an exploratory analysis and selected the group [Quintile 4 (5.8–6.5 mg/dl)] with the lowest risk of mortality as the reference group. Using Cox proportional hazards regression, we performed three multivariate models. Model I was based on age (continuous, years), and sex (male or female). In model II, outcomes were further assessed according to race (Mexican American, other Hispanic, non-Hispanic white, non-Hispanic black, or other (including multi-racial), and education level (<9 years, 9–13 years, or ≥13 years), and poverty-to-income ratio (continuous), BMI (continuous), smoking status (never a smoker, former smoker, and current smoker), comorbidity (diabetes, hypertension, CVD, stroke), hypolipidemic medications (no, other, yes), physical activity (inactive, active), and alcohol intake (none, moderate, heavy) were further adjusted. In model III, the results were adjusted for healthy eating index (continuous), HDL-C (continuous), TC (continuous), and eGFR (continuous). In addition, a two-stage linear regression model with smoothed curves was used to investigate the non-linear relationship between SUA levels and all-cause mortality. Single-linear linear regression was compared with two-slice linear regression using likelihood ratio tests. To determine the threshold value, we selected the point with the highest likelihood among all possible values. A similar approach was applied to assess the association between SUA levels and the risk of all-cause mortality stratified by sex.

We further stratified the analysis by age (<65 or ≥65 years), sex (male or female), race and ethnicity (non-white or other), BMI (<24.9, 25–29.9, ≥30 kg/m^2^), smoking status (never a smoker, former smoker, and current smoker), diabetes (yes or no), hypertension (yes or no), CVD (yes or no), stroke (yes or no), hypolipidemic medications (no, other, yes), physical activity (inactive, active), and alcohol intake (none, moderate, heavy). Interactions were evaluated using the *P*-values for the production terms between SUA and the stratified factors.

We also performed a series of sensitivity analyses. (1) To calculate missing data, we used multiple imputations based on five replications of the Markov-chain Monte Carlo method. (2) To minimize potential reverse causality bias, we excluded participants who died within 2 years of follow-up. (3) Serum TG levels were measured in subjects who were examined only in the morning and those required to fast for at least 8.5 h, but less than 24 h, and LDL-C was calculated based on measurements of total cholesterol (TC), TG, and HDL according to the Friedewald calculation: [LDL-C] = [TC] – [HDL-C] – [TG/5]. Consequently, there were a larger number of missing values for TG and LDL-C (55% and 53%, respectively), and thus to verify the robustness of the results, we further adjusted TG and LDL levels in the model.

All analyses were performed using the R package (version 4.1.3) and Free Statistics software version 1.7, and a two-sided *P*-value <0.05 was set as the threshold for statistical significance.

## Results

### Study participants and baseline characteristics

During a median follow-up of 9.4 years, a total of 2,079 deaths occurred. Among these deaths, the major causes were cardiac and cerebrovascular diseases, accounting for 30.4%, and malignant tumors, accounting for 22.7%. Baseline characteristics of the subjects presented as the quintiles or categories of SUA [Quintile 1 (<4.2 mg/dl), Quintile 2 (4.3–4.9 mg/dl), Quintile 3 (5.0–5.7 mg/dl), Quintile 4 (5.8–6.5 mg/dl), and Quintile 5 (>6.6 mg/dl)] are shown in Table 1. Of the 20,038 patients with hyperlipidemia, those who met the inclusion criteria were identified (Figure 1). As shown in Table 1, participants with a higher SUA level were more likely to be male, non-Hispanic black, and have a higher BMI, and higher rate of smoking, combined with hypertension and CVD, a lower healthy eating index, lower physically activity, higher alcohol intake, lower levels of HDL-C and eGFR, and higher levels of TG, TC, and LDL-C.

**Table 1:** Baseline characteristics of the study participants.

Variables		Serum uric acid level (mg/dl)					*P-*value
		<4.2	4.3–4.9	5.0–5.7	5.8–6.5	>6.6	
	Total (*n* = 20,038)	Quintile1 (*n* = 3,929)	Quintile2 (*n* = 3,747)	Quintile3 (*n* = 4,541)	Quintile4 (*n* = 3,753)	Quintile5 (*n* = 4,068)	
Age, years, *n* (%)							<0.001
<65	16,264 (81.2)	3,253 (82.8)	3,056 (81.6)	3,580 (78.8)	3,024 (80.6)	3,351 (82.4)	
≥65	3,774 (18.8)	676 (17.2)	691 (18.4)	961 (21.2)	729 (19.4)	717 (17.6)	
Age, years, Mean ± SD	48.6 ± 16.4	48.0 ± 16.0	48.9 ± 16.2	49.8 ± 16.6	48.6 ± 16.5	47.4 ± 16.5	<0.001
Sex, *n* (%)							<0.001
Female	9,904 (49.4)	3,340 (85.0)	2,563 (68.4)	2,196 (48.4)	1,129 (30.1)	676 (16.6)	
Male	10,134 (50.6)	589 (15.0)	1,184 (31.6)	2,345 (51.6)	2,624 (69.9)	3,392 (83.4)	
Race-ethnicity, *n* (%)							<0.001
Non-Hispanic White	8,961 (44.7)	1,688 (43)	1,602 (42.8)	2,025 (44.6)	1,773 (47.2)	1,873 (46)	
Non-Hispanic Black	3,383 (16.9)	613 (15.6)	591 (15.8)	762 (16.8)	613 (16.3)	804 (19.8)	
Other Hispanic	1,815 (9.1)	420 (10.7)	371 (9.9)	414 (9.1)	312 (8.3)	298 (7.3)	
Mexican American	3,947 (19.7)	875 (22.3)	828 (22.1)	913 (20.1)	702 (18.7)	629 (15.5)	
Other Race	1,932 (9.6)	333 (8.5)	355 (9.5)	427 (9.4)	353 (9.4)	464 (11.4)	
Poverty Income Ratio, (%)							<0.001
<1.0	4,152 (20.7)	869 (22.1)	803 (21.4)	968 (21.3)	758 (20.2)	754 (18.5)	
1.0–3.0	8,369 (41.8)	1,644 (41.8)	1,594 (42.5)	1,886 (41.5)	1,539 (41.0)	1,706 (41.9)	
≥3.0	7,517 (37.5)	1,416 (36)	1,350 (36)	1,687 (37.2)	1,456 (38.8)	1,608 (39.5)	
Poverty Income Ratio, Mean ± SD	2.5 ± 1.6	2.5 ± 1.6	2.5 ± 1.6	2.5 ± 1.6	2.6 ± 1.6	2.6 ± 1.6	<0.001
Education Level, *n* (%)							<0.001
Low (≤9 years)	2,348 (11.7)	526 (13.4)	475 (12.7)	521 (11.5)	422 (11.2)	404 (9.9)	
Medium (9–13 years)	7,649 (38.2)	1,415 (36.0)	1,420 (37.9)	1,801 (39.7)	1,432 (38.2)	1,581 (38.9)	
High (≥13 years)	10,041 (50.1)	1,988 (50.6)	1,852 (49.4)	2,219 (48.9)	1,899 (50.6)	2,083 (51.2)	
BMI, kg/m^2^, *n* (%)							<0.001
<24.9	4,910 (24.5)	1,528 (38.9)	1,077 (28.7)	1,068 (23.5)	684 (18.2)	553 (13.6)	
25.0–29.9	7,282 (36.3)	1,360 (34.6)	1,358 (36.2)	1,678 (37.0)	1,412 (37.6)	1,474 (36.2)	
≥30	7,846 (39.2)	1,041 (26.5)	1,312 (35)	1,795 (39.5)	1,657 (44.2)	2,041 (50.2)	
BMI, kg/m^2^, Mean ± SD	29.4 ± 6.4	27.2 ± 5.7	28.7 ± 6.2	29.7 ± 6.5	30.2 ± 6.4	31.2 ± 6.6	<0.001
Smoking Status, *n* (%)							<0.001
Never Smoker	10,667 (53.2)	2,352 (59.9)	2,081 (55.5)	2,396 (52.8)	1,870 (49.8)	1,968 (48.4)	
Former Smoker	4,772 (23.8)	701 (17.8)	817 (21.8)	1,103 (24.3)	954 (25.4)	1,197 (29.4)	
Current Smoker	4,599 (23.0)	876 (22.3)	849 (22.7)	1,042 (22.9)	929 (24.8)	903 (22.2)	
**Comorbidity**							
Hypertension, *n* (%)							<0.001
No	12,504 (62.4)	2,711 (69)	2,491 (66.5)	2,820 (62.1)	2,261 (60.2)	2,221 (54.6)	
Yes	7,534 (37.6)	1,218 (31)	1,256 (33.5)	1,721 (37.9)	1,492 (39.8)	1,847 (45.4)	
Diabetes, *n* (%)							0.853
No	16,740 (83.5)	3,295 (83.9)	3,125 (83.4)	3,782 (83.3)	3,123 (83.2)	3,415 (83.9)	
Yes	3,298 (16.5)	634 (16.1)	622 (16.6)	759 (16.7)	630 (16.8)	653 (16.1)	
CVD, *n* (%)							<0.001
No	18,327 (91.5)	3,683 (93.7)	3,461 (92.4)	4,112 (90.6)	3,381 (90.1)	3,690 (90.7)	
Yes	1,711 (8.5)	246 (6.3)	286 (7.6)	429 (9.4)	372 (9.9)	378 (9.3)	
Stroke, *n* (%)							0.127
No	19,468 (97.2)	3,828 (97.4)	3,638 (97.1)	4,390 (96.7)	3,644 (97.1)	3,968 (97.5)	
Yes	570 (2.8)	101 (2.6)	109 (2.9)	151 (3.3)	109 (2.9)	100 (2.5)	
Hypolipidemic Medications, *n* (%)							<0.001
No	9,372 (46.8)	1,668 (42.5)	1,708 (45.6)	2,082 (45.8)	1,823 (48.6)	2,091 (51.4)	
Other*	6,977 (34.8)	1,584 (40.3)	1,360 (36.3)	1,567 (34.5)	1,176 (31.3)	1,290 (31.7)	
Yes	3,689 (18.4)	677 (17.2)	679 (18.1)	892 (19.6)	754 (20.1)	687 (16.9)	
Healthy Eating Index, Mean ± SD	50.3 ± 13.5	50.9 ± 13.9	50.7 ± 13.8	50.7 ± 13.4	49.9 ± 13.2	49.2 ± 13.0	<0.001
Physical Activity, *n* (%)							<0.001
Inactive	7,745 (38.7)	1,706 (43.4)	1,552 (41.4)	1,808 (39.8)	1,326 (35.3)	1,353 (33.3)	
Active	12,293 (61.3)	2,223 (56.6)	2,195 (58.6)	2,733 (60.2)	2,427 (64.7)	2,715 (66.7)	
Alcohol Intake, (%)							<0.001
None	15,246 (76.1)	3,262 (83)	3,042 (81.2)	3,542 (78)	2,710 (72.2)	2,690 (66.1)	
Moderate	1,278 (6.4)	225 (5.7)	225 (6.0)	289 (6.4)	267 (7.1)	272 (6.7)	
Heavy	3,514 (17.5)	442 (11.2)	480 (12.8)	710 (15.6)	776 (20.7)	1,106 (27.2)	
Mortstat, *n* (%)							<0.001
Alive	17,959 (89.6)	3,571 (90.9)	3,384 (90.3)	4,058 (89.4)	3,366 (89.7)	3,580 (88.0)	
Deaths	2,079 (10.4)	358 (9.1)	363 (9.7)	483 (10.6)	387 (10.3)	488 (12.0)	
Follow-up in years, Median (IQR)	9.4 (5.2, 13.3)	9.4 (5.4, 13.6)	9.4 (5.2, 13.3)	9.3 (5.2, 13.2)	9.5 (5.3, 13.3)	9.2 (5.1, 13.3)	0.720
**Laboratory**							
LDL-C, mg/dl, Mean ± SD	126.1 ± 36.5	122.7 ± 34.6	124.9 ± 37.1	126.8 ± 36.8	126.6 ± 37.3	128.8 ± 36.3	<0.001
HDL-C, mg/dl, Mean ± SD	50.2 ± 16.4	56.0 ± 17.6	52.9 ± 17.1	49.8 ± 15.7	47.2 ± 14.8	45.3 ± 14.6	<0.001
TG, mg/dl, Median (IQR)	123.0 (87.0, 180.0)	100.0 (69.0, 147.0)	112.0 (79.0, 160.0)	122.0 (89.0, 174.0)	134.0 (94.0, 191.0)	151.0 (105.0, 215.0)	<0.001
TC, mg/dl, Mean ± SD	207.3 ± 43.1	204.1 ± 42.3	206.5 ± 43.3	207.0 ± 42.8	207.5 ± 43.1	211.4 ± 43.8	<0.001
eGFR, (ml/min/1.73 m^2^), Mean ± SD	95.6 ± 20.7	101.7 ± 19.0	98.0 ± 19.4	94.7 ± 20.4	93.4 ± 20.7	90.7 ± 22.0	<0.001

BMI, body mass index; CVD, cardiovascular disease; TC, total cholesterol; TG, triglyceride; HDL-C, high-density lipoproteincholesterol; LDL-C, low-density lipoprotein-cholesterol; eGFR: estimated glomerular filtration rate.

*Indicated drugs except hypolipidemic medications.

### Multivariate Cox proportional hazards regression analysis of serum uric acid levels and all-cause mortality in patients with hyperlipidemia

Multivariate Cox regression analysis using categorized SUA revealed a non-linear relationship between SUA and all-cause mortality in model III. Compared with group Quintile 4 (5.8–6.5 mg/dl), the HR of Quintile 1 (<4.2 mg/dl), Quintile 2 (4.3–4.9 mg/dl), Quintile 3 (5.0–5.7 mg/dl), and Quintile 5 (>6.6 mg/dl) were 1.24 (95% CI: 1.06–1.45), 1.19 (95% CI: 1.03–1.38), 1.07 (95% CI: 0.94–1.23), and 1.29 (95%CI: 1.13–1.48), respectively, after adjusting for all covariates in model III (Table 2).

### Non-linear relationship between serum uric acid and all-cause mortality of hyperlipidemia

**Figure 2:**
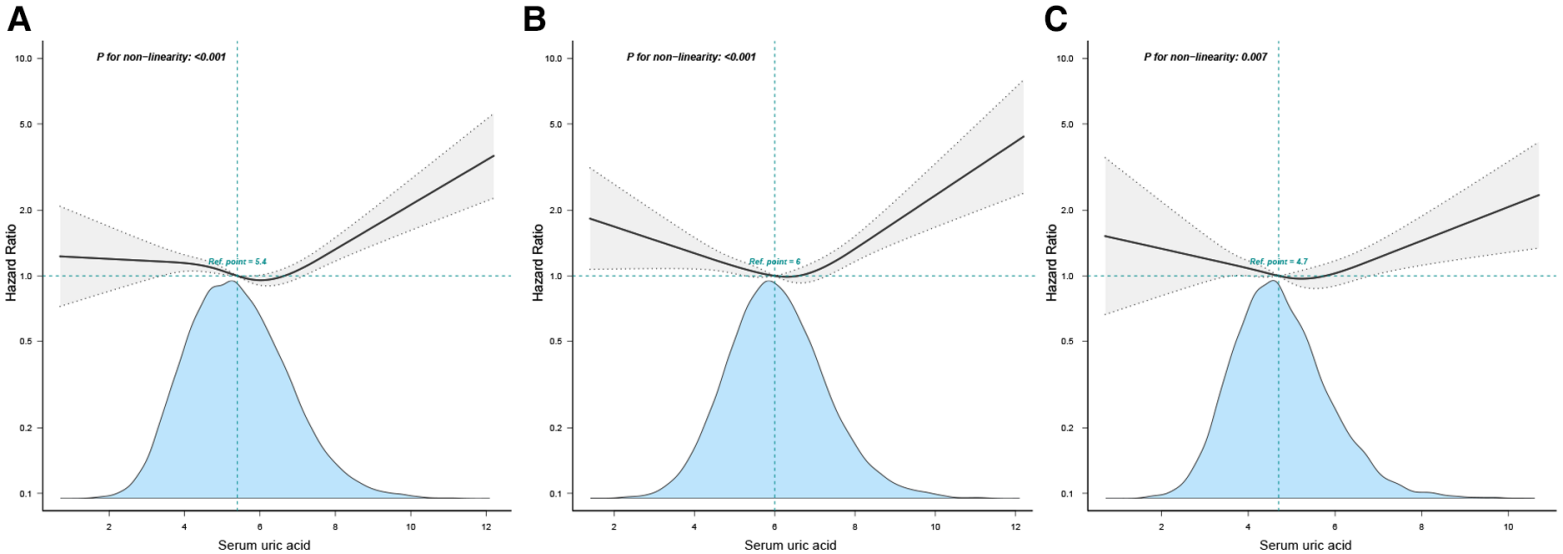
Multivariable-adjusted hazard ratios for all-cause mortality by serum uric acid level: (**A**) all population, (**B**) male, and (**C**) female. Age, sex, race, education, BMI, poverty income ratio, smoking status, diabetes, hypertension, CVD, stroke, hypolipidemic medications, physical activity, alcohol intake, HDL-C, TC, eGFR, healthy eating index were adjusted. The black and dotted lines represent the estimated values and their corresponding 95% confidence intervals, respectively.

Using a multivariate Cox regression model and a smoothed curve fit, we observed a non-linear relationship between SUA levels and all-cause mortality in the entire study population, and in both male and female populations (Figure 2). A pairwise multivariate Cox regression model was fitted to the data to obtain two different slopes. We used a two-slice model to fit the association between SUA levels and mortality, where the *P*-value of the log-likelihood ratio test was <0.001 in both the entire population and the male or female population (Table 3). For the entire population, to the left side of the inflection point (6.3 mg/dl), the HR was 0.91 (95% CI: 0.85–0.97), and to right side of the inflection point, the HR was 1.22 (95% CI: 1.10–1.35). For women, the HR on the left side of the inflection point (6.0 mg/dl) was 0.91 (95% CI: 0.83–1.00), and that on the right side was 1.41 (95% CI: 1.16–1.73). Among men, the HR on the left side of the inflection point (6.5 mg/dl) was 0.92 (95% CI: 0.84–1.00), whereas that on the right side was 1.30 (95% CI: 1.14–1.48).

**Table 2:** Multivariate Cox regression for serum uric acid on all-cause mortality of hyperlipidemia.

Variable	Deaths/Total	Nonadjusted Model	*P-*value	Model I	*P-*value	Model II	*P-*value	Model III	*P-*value
	N	HR (95% CI)		HR (95% CI)		HR (95% CI)		HR (95% CI)	
**Continuous variable**									
Serum uric acid level (mg/dl)	2,079/20,038	1.09 (1.06–1.13)	<0.001	1.06 (1.02–1.10)	0.001	1.05 (1.01–1.09)	0.011	1.03 (0.99–1.08)	0.092
**Binary variable**									
Quintile 1 (<4.2)	358/3,929	0.88 (0.76–1.01)	0.074	1.14 (0.98–1.33)	0.080	1.19 (1.02–1.39)	0.026	1.24 (1.06–1.45)	0.007
Quintile 2 (4.3–4.9)	363/3,747	0.94 (0.82–1.09)	0.419	1.09 (0.94–1.26)	0.250	1.17 (1.01–1.35)	0.04	1.19 (1.03–1.38)	0.022
Quintile 3 (5.0–5.7)	483/4,541	1.04 (0.91–1.19)	0.572	1.03 (0.9–1.17)	0.704	1.06 (0.93–1.22)	0.372	1.07 (0.94–1.23)	0.306
Quintile 4 (5.8–6.5)	387/3,753	Ref.		Ref.		Ref.		Ref.	
Quintile 5 (>6.6)	488/4,068	1.18 (1.03–1.34)	0.018	1.28 (1.12–1.46)	<0.001	1.32 (1.16–1.51)	<0.001	1.29 (1.13–1.48)	<0.001
P for trend	* *		<0.001		0.246		0.513		0.818

Model I: adjusted for age + sex; Model II: adjusted for Model I + race + education + BMI + poverty income ratio + smoking status + diabetes + hypertension + CVD + stroke + hypolipidemic medications + physical activity + alcohol intake; Model III: adjusted for Model II + HDL-C + TC + eGFR + healthy eating index.

SUA, serum uric acid; BMI, body mass index; CVD, cardiovascular disease; TC, total cholesterol; TG, triglyceride; HDL-C, high-density lipoproteincholesterol; LDL-C, low-density lipoprotein-cholesterol; eGFR: estimated glomerular filtration rate.

**Table 3:** Threshold effect analysis of serum uric acid on all-cause mortality by sex

Total population	HR	95% CI	*P-*value
Serum uric acid (mg/dl)	1.03	0.99–1.08	0.092
Inflection point			
<6.3	0.91	0.85–0.97	0.003
≥6.3	1.22	1.10–1.35	<0.001
P for log-likelihood ratio test			<0.001
**Male**			
Serum uric acid (mg/dl)	1.05	0.99–1.11	0.053
Inflection point			
<6.5	0.92	0.84–1.00	0.054
≥6.5	1.30	1.14–1.48	<0.001
P for log-likelihood ratio test			<0.001
**Female**			
Serum uric acid (mg/dl)	1.02	0.96–1.09	0.566
Inflection point			
<6.0	0.91	0.83–1.00	0.074
≥6.0	1.41	1.16–1.73	<0.001
P for log-likelihood ratio test			<0.001

Adjusted for age + sex + race + education + BMI + poverty income ratio + smoking status + diabetes + hypertension + CVD + stroke + hypolipidemic medications + physical activity + alcohol intake + HDL-C + TC + eGFR + healthy eating index.

BMI, body mass index; CVD, cardiovascular disease; TC, total cholesterol; TG, triglyceride; HDL-C, high-density lipoproteincholesterol; LDL-C, low-density lipoprotein-cholesterol; eGFR: estimated glomerular filtration rate.

**Table 4:** Associations between serum uric acid with all-cause mortality in various subgroups among patients with hyperlipidemia.

Subgroups		Serum uric acid level (mg/dl)					P interaction
	Deaths/Total	Quintile 1 (<4.2)	Quintile 2 (4.3–4.9)	Quintile 3 (5.0–5.7)	Quintile 4 (5.8–6.5)	Quintile 5 (>6.6)	
	*n*	*n* = 3,929	*n* = 3,747	*n* = 4,541	*n* = 3,753	*n* = 4,068	
Age, years							0.094
<65	790/16,264	0.96 (0.74–1.24)	1.03 (0.81–1.31)	1.03 (0.83–1.29)	Ref.	1.25 (1.01–1.55)	
≥65	1,289/3,774	1.44 (1.18–1.75)	1.30 (1.07–1.57)	1.09 (0.92–1.3)	Ref.	1.30 (1.09–1.55)	
Sex							0.483
Male	1,186/10,134	1.27 (1.01–1.60)	1.24 (1.02–1.52)	1.10 (0.93–1.31)	Ref.	1.28 (1.09–1.51)	
Female	893/9,904	1.20 (0.95–1.52)	1.13 (0.89–1.43)	1.01 (0.8–1.27)	Ref.	1.40 (1.08–1.82)	
Race-ethnicity							0.969
Non-Hispanic White	1,454/8,961	1.19 (0.97–1.46)	1.13 (0.93–1.37)	1.02 (0.86–1.22)	Ref.	1.32 (1.11–1.58)	
Other	825/11,077	1.30 (1.01–1.67)	1.29 (1.01–1.63)	1.15 (0.92–1.43)	Ref.	1.25 (1.01–1.56)	
BMI, kg/m^2^							0.754
<24.9	635/4,910	1.50 (1.12–2.01)	1.43 (1.08–1.91)	1.30 (0.99–1.70)	Ref.	1.69 (1.26–2.27)	
25.0–29.9	818/7,282	1.13 (0.87–1.46)	1.09 (0.86–1.38)	1.01 (0.81–1.25)	Ref.	1.16 (0.93–1.45)	
≥30	626/7,846	1.17 (0.86–1.58)	1.16 (0.88–1.53)	1.01 (0.79–1.27)	Ref.	1.23 (0.98–1.54)	
Smoking Status							0.478
Never Smoker	778/10,667	1.52 (1.18–1.97)	1.27 (0.99–1.64)	1.18 (0.94–1.50)	Ref.	1.39 (1.09–1.77)	
Former Smoker	771/4,772	1.25 (0.95–1.64)	1.19 (0.92–1.53)	1.17 (0.94–1.46)	Ref.	1.30 (1.04–1.61)	
Current Smoker	530/4,599	0.94 (0.69–1.27)	1.10 (0.83–1.45)	0.86 (0.66–1.11)	Ref.	1.11 (0.85–1.46)	
Hypertension							0.116
Yes	1,350/7,534	1.19 (0.97–1.45)	1.34 (1.11–1.61)	1.13 (0.95–1.33)	Ref.	1.39 (1.18–1.64)	
No	729/12,504	1.32 (1.02–1.70)	0.98 (0.77–1.26)	0.96 (0.76–1.21)	Ref.	1.10 (0.87–1.41)	
Diabetes							0.824
Yes	615/3,298	1.19 (0.90–1.58)	1.21 (0.92–1.59)	1.07 (0.83–1.38)	Ref.	1.35 (1.05–1.73)	
No	1,464/16,740	1.26 (1.04–1.53)	1.17 (0.97–1.40)	1.08 (0.92–1.27)	Ref.	1.27 (1.08–1.50)	
CVD							0.723
Yes	513/1,711	1.02 (0.73–1.43)	1.52 (1.13–2.04)	1.13 (0.87–1.48)	Ref.	1.40 (1.07–1.83)	
No	1,566/18,327	1.27 (1.06–1.53)	1.10 (0.92–1.31)	1.05 (0.89–1.23)	Ref.	1.25 (1.07–1.47)	
Stroke							0.318
Yes	171/570	0.81 (0.45–1.45)	1.22 (0.73–2.03)	1.04 (0.64–1.69)	Ref.	1.22 (0.72–2.09)	
No	1,908/19,468	1.28 (1.09–1.51)	1.18 (1.01–1.38)	1.09 (0.94–1.25)	Ref.	1.30 (1.13–1.50)	
CKD							0.747
Yes	429/1,063	1.01 (0.65–1.54)	1.02 (0.71–1.46)	0.98 (0.72–1.32)	Ref.	1.11 (0.85–1.46)	
No	1,650/18,975	1.31 (1.10–1.56)	1.25 (1.06–1.47)	1.11 (0.95–1.29)	Ref.	1.35 (1.15–1.59)	
Hypolipidemic Medications							0.578
No	490/9,372	1.09 (0.78–1.53)	0.96 (0.71–1.3)	0.87 (0.66–1.16)	Ref.	1.19 (0.91–1.57)	
Other	881/6,977	1.22 (0.96–1.56)	1.31 (1.04–1.66)	1.02 (0.82–1.27)	Ref.	1.26 (1.02–1.57)	
Yes	708/3,689	1.36 (1.05–1.78)	1.18 (0.91–1.52)	1.24 (0.99–1.55)	Ref.	1.40 (1.10–1.77)	
Physical Activity							0.861
Inactive	978/7,745	1.18 (0.94–1.47)	1.12 (0.91–1.39)	1.00 (0.82–1.21)	Ref.	1.26 (1.03–1.53)	
Active	1,101/12,293	1.33 (1.06–1.66)	1.28 (1.04–1.57)	1.17 (0.97–1.42)	Ref.	1.33 (1.10–1.60)	
Alcohol intake							0.520
None	1,653/15,246	1.31 (1.10–1.55)	1.19 (1.00–1.40)	1.13 (0.97–1.31)	Ref.	1.27 (1.09–1.49)	
Moderate	123/1,278	1.17 (0.55–2.51)	1.78 (0.92–3.44)	1.00 (0.54–1.89)	Ref.	1.45 (0.78–2.70)	
Heavy	303/3,514	0.92 (0.58–1.47)	1.13 (0.75–1.69)	0.79 (0.55–1.14)	Ref.	1.28 (0.93–1.75)	

Adjusted for age + sex + race + education + BMI + poverty income ratio + smoking status + diabetes + hypertension + CVD + stroke + hypolipidemic medications + physical activity + alcohol intake + HDL-C + TC + eGFR + healthy eating index. The strata variable was not included when stratifying by itself.

BMI, body mass index; CVD, cardiovascular disease; TC, total cholesterol; TG, triglyceride; HDL-C, high-density lipoproteincholesterol; LDL-C, low-density lipoprotein-cholesterol; eGFR: estimated glomerular filtration rate.

### Stratified and sensitivity analyses

On the basis of stratified analysis, we found no evidence to indicate that the U-shaped relationship between SUA and all-cause mortality changed with age (<65 or ≥65 years), sex (male or female), race and ethnicity (non-white or other), BMI (<24.9, 25–29.9, ≥30 kg/m^2^), smoking status (never a smoker, former smoker, and current smoker), diabetes (yes or no), hypertension (yes or no), CVD (yes or no), stroke (yes or no), hypolipidemic medications (no, other, yes), physical activity (inactive, active), or alcohol intake (none, moderate, heavy) (Table 4). Moreover, we detected no significant interactions among any of the strata of variables.

Although we also performed multiple imputation sensitivity analysis, this did not significantly alter the aforementioned results. (Supplementary material Table S1). Having excluded those participants who had died within 2 years of follow-up, the results in our sensitivity analyses were generally robust (Supplementary material Table S2), and with further adjusting for TG and LDL-C (Supplementary material Table S3).

## Discussion

In this study, we assessed the optimal levels of SUA in patients with hyperlipidemia recruited from a nationally representative US population dataset. In this cohort, SUA showed a U-shaped relationship with all-cause mortality in both males and females. Threshold effect analysis revealed that overall, an average SUA level of 6.3 mg/dl (6.5 mg/dl in males and 6.0 mg/dl in females) may be considered safe with respect to patient survival.

A recent review of studies conducted on populations with different health conditions (9), including healthy individuals (16, 17), patients with coronary heart disease (18), older patients (19), patients with hypertension (20), and patients with chronic kidney disease (21), summarized the U-shaped association between SUA and various adverse clinical outcomes, such as all-cause mortality and cardiovascular mortality. Our study confirmed the U-shaped relationship between SUA and all-cause mortality in patients with hyperlipidemia. It should be noted, however, that although these studies have confirmed a U-shaped relationship between SUA and all-cause mortality, the authors reported different optimal thresholds for SUA. For example, Weicheng et al. found that in an older population aged >65 years, both low (<4 mg/dl) and high (>8 mg/dl) SUA levels were independently associated with an increased risk of all-cause mortality (19), and similarly in a cohort study of a Chinese population with coronary artery disease, the authors identified a U-shaped association in which the risk of all-cause mortality increased at both low (≤5.05 mg/dl) and high (≥8.0 mg/dl) levels of SUA (18). In addition, a cohort study including 375,163 Korean general medical examination population found that both low uric acid group (<3.5 mg/dl in men; <2.5 mg/dl in women) and high uric acid group (>9.5 mg/dl in men; >8.5 mg/dl in women) increased all-cause mortality compared to the sex-specific reference group (22). Our analyses in the present study indicate that the optimal SUA level for hyperlipidemic patients is 6.5 mg/dl in males and 6.0 mg/dl in females, and that levels either above or below these thresholds are associated with an increased risk of all-cause mortality. We suspect that differences in the values reported by different studies may be partially attributable to differences in one or more of the following factors: subjects, clinical characteristics, sample size, grouping strategy, and adjustment for confounding factors. Furthermore, the findings of the Fremantle Diabetes Study indicated that SUA was not an independent predictor of cardiovascular disease-associated mortality or all-cause mortality in patients with type 2 diabetes (23), and consistently, the findings of two other large population-based observational studies conducted in Korea (24) and the United States (11) indicated no significant correlation between SUA and all-cause mortality or cardiovascular mortality. In this regard, it is, however, noteworthy that individuals who had taken uric acid-lowering drugs and diuretics that affect uric acid levels were not excluded from these aforementioned studies. Consequently, the inclusion of these patients may have obscured the actual association between uric acid and all-cause mortality. In contrast, in this study, we excluded those who were pregnant at baseline, had used uric acid-lowering drugs and diuretics within 1 month, had an eGFR values <30 ml/min/1.73 m^2^ at baseline, and had cancer, we also took into account the effect of using lipid-lowering drugs on uric acid levels, and stratified our analysis according to those who did and did not use these drugs. We accordingly detected a consistent U-shaped association between uric acid and all-cause mortality.

Although the pathophysiological role of uric acid has been studied and debated for decades with respect to a range of disease processes, we have yet to gain a complete understanding of its multiple effects (9). Hyper-uric acid levels have been used as an independent risk factor for adverse clinical outcomes, and it is well established that atherosclerosis is a major cause of death in patients with hyperlipidemia (25), High levels of uric acid are associated with and contribute to the atherosclerotic process, and studies have demonstrated that SUA maintains the atherosclerotic state by interfering with lipid metabolism, reducing endothelial cell nitric oxide synthesis, promoting the proliferation of vascular smooth muscle cells, and overcoming the inflammatory response process (26). Moreover, hyperuricemia is believed to be a mediator promoting pro-inflammatory endocrine imbalance in adipose tissues, which may be an important factor in the development of dyslipidemia and the inflammatory processes leading to the development of atherosclerosis (27). Consistently, in the present study, we found that higher uric acid levels were associated with higher levels of TG, TC, and LDL-C and lower HDL-C levels, and that hyper-uric acid is associated with an increase in the mortality of patients with hyperlipidemia, even after adjusting for common cardiovascular risk factors such as LDL-C, TG, and TC.

Although much of the previous research has focused on the pathophysiological effects of high levels of uric acid (hyperuricemia), more recently, the focus of research has extended to examining the effects of hypouricemia, with the findings of several studies indicating that abnormally low levels of SUA may have potentially severe effects. In this regard, it has been suggested that SUA may act as an important antioxidant molecule, accounting for 50% of the body's total antioxidant capacity (7), and it has been demonstrated that low SUA levels are associated with a loss of free radical scavenging capacity, which may in turn contribute to an increase in vascular endothelial cell damage and thus higher cardiovascular-associated mortality (18). Similarly, in a retrospective cohort study of hypertensive patients, low values of uric acid were found to be associated with an increase in cardiovascular mortality, and is potentially a causal factor for stroke and heart-specific mortality (20). Furthermore, the findings of a recent study have indicated that malnutrition is an important factor contributing to higher CVD-related mortality in older patients with low levels of uric acid (19), with older patients with more severe malnutrition showing higher levels of hyponatremia-related mortality. Consistently, it has been established that low serum uric acid concentrations (< 2.5 mg/dl in women and <3.5 mg/dl in men) are associated with a low BMI (<18.5 kg/m^2^) and higher rates of weight loss (28). Likewise, in the present study, we found that individuals with a lower body mass index were more likely to have low serum levels of uric acid, which may serve as a predictor of malnutrition. However, in our stratified analysis of BMI, we found that low uric acid was associated with higher all-cause mortality in each stratum, possibly indicating an independent effect of low uric acid levels on all-cause mortality. However, we did not further analyze the association between uric acid and cardiac-specific mortality to verify whether cardiac death was attributable to the increase in all-cause mortality associated with these low levels of uric acid. Consistently, a further study of a US population with low uric acid levels found that this condition was associated with all-cause mortality in men (29), and possibly contributed to an increase in diabetes-related mortality. In this context, hyperglycemia is assumed to enhances uric acid excretion and prolonged hyperglycemia can lead to uremia, resulting in hypouricemia in diabetics. In cases of severe diabetes, hyperglycemia may lead to extreme hypouricemia (analogous to reverse causality) (30). However, the U-shaped association between uric acid levels and all-cause mortality obtained in our stratified analysis was identified for both diabetic and non-diabetic populations, thereby providing possible evidence to indicate an independent association between low values of uric acid and all-cause mortality, which is not necessarily mediated exclusively via the aforementioned pathways.

Although the findings of this study provide important insights with regards to serum uric acid levels, several limitations of the study need to be considered when interpreting these findings. Firstly, this was retrospective cohort study, and thus we were unable to establish causal inferences. Secondly, although we excluded factors affecting uric acid as much as possible, it cannot be conclusively established that factors that might potentially influence uric acid levels were completely excluded. For example, certain antihypertensive drugs, such as angiotensin receptor blockers, may effect uric acid levels. Nevertheless, to minimize bias, we performed sensitivity analysis to further confirm that our results were consistent for individuals with and without hypertension. Thirdly, although we adjusted for multiple potential confounders, we have not been able to completely exclude the possibility of residual confounding effects attributable to unmeasured factors. Furthermore, SUA levels were measured at a single point in time, and consequently, were unable to establish any temporal associations between SUA levels and any of the assessed disease markers. However, despite these limitations, we believe that this study has certain strengths. Firstly, we used a nationally representative sample, which facilitated generalization of the findings to the U.S. population as a whole, with a long-term follow-up period of up to 9 years and a low incidence of mismatched records in the NHANES-associated mortality file. Secondly, given the benefit of the comprehensive data collected in NHANES, we were able to control for potential confounding effects associated with a range of demographic, socioeconomic, lifestyle, and dietary factors. Thirdly, in an effort to minimize the bias associated with missing data, we performed multiple interpolation, whereas in the sensitivity analysis, we further adjusted for LDL-C and TG, which were not included in the model owing to a large amount of missing data, and found that the results remained consistent.

In conclusion, we established that both low and high serum levels of uric acid were significantly associated with an increased risk of all-cause mortality, both in the overall US hyperlipidemic population and for the two sexes. These findings will contribute to establishing a target value for uric acid-lowering therapy in the treatment of hyperlipidemic patients.

## Data Availability

The raw data supporting the conclusions of this article will be made available by the authors, without undue reservation.

## References

[1] Yao YS Li TD Zeng ZH. Mechanisms underlying direct actions of hyperlipidemia on myocardium: an updated review. Lipids Health Dis. (2020) 19(1):23. 10.1186/s12944-019-1171-8 32035485 PMC7007679

[2] Hill MF Bordoni B. Hyperlipidemia. StatPearls. Treasure Island, FL: StatPearls Publishing (2022). Copyright © 2022, StatPearls Publishing LLC. 32644608

[3] Alloubani A Nimer R Samara R. Relationship between hyperlipidemia, cardiovascular disease and stroke: a systematic review. Curr Cardiol Rev. (2021) 17(6):e051121189015. 10.2174/1573403X16999201210200342 33305711 PMC8950504

[4] Ali N Rahman S Islam S Haque T Molla NH Sumon AH The relationship between serum uric acid and lipid profile in Bangladeshi adults. BMC Cardiovasc Disord. (2019) 19(1):42. 10.1186/s12872-019-1026-2 30791868 PMC6385393

[5] Schlotte V Sevanian A Hochstein P Weithmann KU. Effect of uric acid and chemical analogues on oxidation of human low density lipoprotein in vitro. Free Radical Biol Med. (1998) 25(7):839–47. 10.1016/S0891-5849(98)00160-9 9823550

[6] Becker BF. Towards the physiological function of uric acid. Free Radical Biol Med. (1993) 14(6):615–31. 10.1016/0891-5849(93)90143-I 8325534

[7] Ndrepepa G. Uric acid and cardiovascular disease. Clin Chim Acta. (2018) 484:150–63. 10.1016/j.cca.2018.05.046 29803897

[8] Ames BN Cathcart R Schwiers E Hochstein P. Uric acid provides an antioxidant defense in humans against oxidant- and radical-caused aging and cancer: a hypothesis. Proc Natl Acad Sci U S A. (1981) 78(11):6858–62. 10.1073/pnas.78.11.6858 6947260 PMC349151

[9] Crawley WT Jungels CG Stenmark KR Fini MA. U-shaped association of uric acid to overall-cause mortality and its impact on clinical management of hyperuricemia. Redox Biol. (2022) 51:102271. 10.1016/j.redox.2022.102271 35228125 PMC8889273

[10] Stack AG Hanley A Casserly LF Cronin CJ Abdalla AA Kiernan TJ Independent and conjoint associations of gout and hyperuricaemia with total and cardiovascular mortality. QJM - Mon J Assoc Physicians. (2013) 106(7):647–58. 10.1093/qjmed/hct083 23564632

[11] Zalawadiya SK Veeranna V Mallikethi-Reddy S Bavishi C Lunagaria A Kottam A Uric acid and cardiovascular disease risk reclassification: findings from NHANES III. Eur J Prev Cardiol. (2015) 22(4):513–8. 10.1177/2047487313519346 24431384

[12] Li B Chen L Hu X Tan T Yang J Bao W Association of serum uric acid with all-cause and cardiovascular mortality in diabetes. Diabetes Care. (2023) 46(2):425–33. 10.2337/dc22-1339 36490263

[13] Third report of the national cholesterol education program (NCEP) expert panel on detection, evaluation, and treatment of high blood cholesterol in adults (adult treatment panel III) final report. Circulation. (2002) 106(25):3143–421. 10.1161/circ.106.25.3143 12485966

[14] Sarah SC Fradkin JE Saydah SH Rust KF Cowie CC. The prevalence of meeting A1C, blood pressure, and LDL goals among people with diabetes, 1988–2010. Diabetes Care. (2013) 36(8):2271–9. 10.2337/dc12-2258 23418368 PMC3714503

[15] Liu Y Geng T Wan Z Lu Q Zhang X Qiu Z Associations of serum folate and vitamin B12 levels with cardiovascular disease mortality among patients with type 2 diabetes. JAMA Network Open. (2022) 5(1):e2146124. 10.1001/jamanetworkopen.2021.46124 35099545 PMC8804919

[16] Hu L Hu G Xu BP Zhu L Zhou W Wang T U-shaped association of serum uric acid with all-cause and cause-specific mortality in US adults: a cohort study. J Clin Endocrinol Metab. (2020) 105(3):e597–e609. 10.1210/clinem/dgz068 31650159

[17] Zhang S Liu L Huang YQ Lo K Feng YQ. A U-shaped association between serum uric acid with all-cause mortality in normal-weight population. Postgrad Med. (2020) 132(4):391–7. 10.1080/00325481.2020.1730610 32098577

[18] Zheng Y Ou J Huang D Zhou Z Dong X Chen J The U-shaped relationship between serum uric acid and long-term all-cause mortality in coronary artery disease patients: a cohort study of 33,034 patients. Front Cardiovasc Med. (2022) 9:858889. 10.3389/fcvm.2022.858889 35811724 PMC9256977

[19] Tseng WC Chen YT Ou SM Shih CJ Tarng DC. U-shaped association between serum uric acid levels with cardiovascular and all-cause mortality in the elderly: the role of malnourishment. J Am Heart Assoc. (2018) 7(4):e007523. 10.1161/JAHA.117.007523 29440009 PMC5850189

[20] You H Chen K Han P Yue C Zhao X. U-sShaped relationship between cardiovascular mortality and serum uric acid may be attributed to stroke- and heart-specific mortality, respectively, among hypertensive patients: a nationally representative cohort study. Med Sci Monit. (2021) 27:e928937. 10.12659/msm.928937 33534782 PMC7869412

[21] Srivastava A Kaze AD McMullan CJ Isakova T Waikar SS. Uric acid and the risks of kidney failure and death in individuals with CKD. Am J Kidney Dis. (2018) 71(3):362–70. 10.1053/j.ajkd.2017.08.017 29132945 PMC5828916

[22] Cho SK Chang Y Kim I Ryu S. U-shaped association between serum uric acid level and risk of mortality: a cohort study. Arthritis Rheumatol. (2018) 70(7):1122–32. 10.1002/art.40472 29694719

[23] Ong G Davis WA Davis TM. Serum uric acid does not predict cardiovascular or all-cause mortality in type 2 diabetes: the fremantle diabetes study. Diabetologia. (2010) 53(7):1288–94. 10.1007/s00125-010-1735-7 20349345

[24] Cheong E Ryu S Lee JY Lee SH Sung JW Cho DS Association between serum uric acid and cardiovascular mortality and all-cause mortality: a cohort study. J Hypertens. (2017) 35(Suppl 1):S3–s9. 10.1097/HJH.0000000000001330 28350618

[25] Libby P Buring JE Badimon L Hansson GK Deanfield J Bittencourt MS Atherosclerosis. Nat Rev Dis Primers. (2019) 5(1):56. 10.1038/s41572-019-0106-z 31420554

[26] Jayachandran M Qu S. Harnessing hyperuricemia to atherosclerosis and understanding its mechanistic dependence. Med Res Rev. (2021) 41(1):616–29. 10.1002/med.21742 33084092

[27] Baldwin W McRae S Marek G Wymer D Pannu V Baylis C Hyperuricemia as a mediator of the proinflammatory endocrine imbalance in the adipose tissue in a murine model of the metabolic syndrome. Diabetes. (2011) 60(4):1258–69. 10.2337/db10-0916 21346177 PMC3064099

[28] Baker JF Weber DR Neogi T George MD Long J Helget LN Associations between low serum urate, body composition, and mortality. Arthritis Rheumatol. (2023) 75(1):133–40. 10.1002/art.42301 35974440 PMC10600587

[29] D’Silva KM Yokose C Lu N McCormick N Lee H Zhang Y Hypouricemia and mortality risk in the US general population. Arthritis Care Res (Hoboken). (2021) 73(8):1171–9. 10.1002/acr.24476 33026684 PMC8091104

[30] Herman JB Goldbourt U. Uric acid and diabetes: observations in a population study. Lancet. (1982) 2(8292):240–3. 10.1016/S0140-6736(82)90324-5 6124672

