# Supplementary material for: U-shaped association of serum uric acid with all-cause mortality in patients with hyperlipidemia in the United States: a cohort study
Source: Front Cardiovasc Med. 2023 May 23;10:1165338. doi: 10.3389/fcvm.2023.1165338 (PMC10242664; doi:10.3389/fcvm.2023.1165338)
Supplement: Supplementary file 1 [file Datasheet1.docx]

Supplementary Material

U-shaped association of serum uric acid with all-cause mortality in patients with hyperlipidemia in the United States: A cohort study

Lihua Huang^1†^, Zhanpeng Lu^2†^, Xiaoyan You^1^, Chunsheng Zhou^1^, Liuliu He^1^, Jingxiang Xie^1^, Xiaoqing Zhou^3*^

***Correspondence: Xiaoqing Zhou: tarzan528@163.com**

# Supplementary Figures and Tables

**eTable 1.** Comparison between the findings obtained from multiple imputation data and those from complete data, based on a mutually adjusted model

| **Variable** | **Complete data** | | **Multiple imputation data** | |
| --- | --- | --- | --- | --- |
|  | **n** | **HR (95% CI)** | **n** | **HR (95% CI)** |
| Age, years | 23493 | 1.08 (1.08~1.09) | 23493 | 1.08 (1.08~1.09) |
| Sex |  |  |  |  |
| Female | 11686 | Ref. | 11686 | Ref. |
| Male | 11807 | 1.47 (1.32~1.63) | 11807 | 1.43 (1.30~1.57) |
| Race-ethnicity |  |  |  |  |
| Non-Hispanic White | 9996 | Ref. | 9996 | Ref. |
| Non-Hispanic Black | 4034 | 0.87 (0.76~0.99) | 4034 | 0.89 (0.79~1.00) |
| Other Hispanic | 2284 | 0.61 (0.50~0.75) | 2284 | 0.59 (0.50~0.71) |
| Mexican American | 4693 | 0.58 (0.50~0.66) | 4693 | 0.58 (0.51~0.66) |
| Other Race | 2486 | 0.72 (0.57~0.90) | 2486 | 0.67 (0.55~0.82) |
| Poverty Income Ratio | 21538 | 0.85 (0.82~0.88) | 23493 | 0.85 (0.82~0.87) |
| Education Level, n (%) |  |  |  |  |
| Low (≤9 years) | 3044 | Ref. | 3049 | Ref. |
| Medium (9–13 years) | 8968 | 1.15 (1.01~1.31) | 8982 | 1.08 (0.96~1.21) |
| High (≥13 years) | 11445 | 1.02 (0.89~1.17) | 11462 | 1.01 (0.89~1.14) |
| BMI, kg/m^2^ | 23157 | 0.99 (0.98~1.00) | 23493 | 0.99 (0.98~0.99) |
| Smoking Status |  |  |  |  |
| Former Smoker | 5465 | 1.24 (1.12~1.38) | 5471 | 1.24 (1.12~1.38) |
| Never a Smoker | 12627 | Ref. | 12639 | Ref. |
| Current Smoker | 5377 | 2.18 (1.93~2.46) | 5383 | 2.18 (1.93~2.46) |
| Hypertension, n (%) |  |  |  |  |
| No | 14586 | Ref. | 14586 | Ref. |
| Yes | 8907 | 1.26 (1.14~1.39) | 8907 | 1.2 (1.10~1.31) |
| Diabetes, n (%) |  |  |  |  |
| No | 19484 | Ref. | 19785 | Ref. |
| Yes | 4008 | 1.47 (1.33~1.63) | 4008 | 1.42 (1.30~1.56) |
| CVD, n (%) |  |  |  |  |
| No | 21451 | Ref. | 21454 | Ref. |
| Yes | 2038 | 1.34 (1.18~1.52) | 2039 | 1.34 (1.19~1.50) |
| Stroke, n (%) |  |  |  |  |
| No | 22777 | Ref. | 22802 | Ref. |
| Yes | 690 | 1.12 (0.93~1.35) | 691 | 1.22 (1.04~1.44) |
| Hypolipidemic Medications, n (%) |  |  |  |  |
| No | 11009 | Ref. | 11022 | Ref. |
| Other | 8106 | 1.19 (1.06~1.34) | 8116 | 1.21 (1.09~1.35) |
| Yes | 4350 | 1.05 (0.91~1.21) | 4355 | 1.05 (0.92~1.20) |
| Physical Activity, n (%) |  |  |  |  |
| Inactive | 9587 | Ref. | 9592 | Ref. |
| Active | 13894 | 0.76 (0.69~0.83) | 13901 | 0.74 (0.68~0.80) |
| Alcohol Intake, % |  |  |  |  |
| None | 16853 | Ref. | 17949 | Ref. |
| Moderate | 1402 | 0.94 (0.78~1.13) | 1494 | 0.86 (0.72~1.03) |
| Heavy | 3803 | 1.08 (0.95~1.24) | 4050 | 1.09 (0.96~1.23) |
| Healthy Eating Index | 22058 | 0.995 (0.992~0.998) | 23493 | 0.995 (0.992~0.998) |
| eGFR, (mL/min/1.73 m^2^) | 23493 | 0.996 (0.993~0.999) | 23493 | 0.995 (0.992~0.998) |
| HDL-C, mg/dL | 23492 | 0.999 (0.996~1.002) | 23493 | 0.999 (0.996~1.002) |
| TC, mg/dL | 23492 | 1.000 (0.999~1.001) | 23493 | 1.000 (0.999~1.001) |
| Serum Uric Acid Level (mg/dL) |  |  |  |  |
| Quintile 1 (<4.2) | 4665 | 1.24 (1.06~1.45) | 4665 | 1.28 (1.11~1.47) |
| Quintile 2 (4.3–4.9) | 4387 | 1.19 (1.03~1.38) | 4387 | 1.19 (1.04~1.36) |
| Quintile 3 (5.0–5.7) | 5287 | 1.07 (0.94~1.23) | 5287 | 1.12 (0.99~1.26) |
| Quintile 4 (5.8–6.5) | 4377 | Ref. | 4377 | Ref. |
| Quintile 5 (>6.6) | 4777 | 1.29 (1.13~1.48) | 4777 | 1.29 (1.14~1.46) |

**Notes:** The model was adjusted for age + sex+ race + education + BMI + poverty income ratio + smoking status + diabetes + hypertension + CVD + stroke+ medication usage + physical activity + alcohol use + HDL-C + TC + eGFR + healthy eating index

**Abbreviations:** BMI, body mass index; CVD, cardiovascular disease; TC, total cholesterol; HDL-C, high-density lipoprotein cholesterol; eGFR: estimated glomerular filtration rate

**eTable 2.** Hazard ratios (95% CIs) of all-cause mortality according to serum uric acid levels after excluding hyperlipidemia patients with less than 2 years of follow-up

| **Variable** | **Deaths/Total** | **Adjusted Model** | ***P-value*** |
| --- | --- | --- | --- |
|  | **n** | **HR (95% CI)** |  |
| Binary variable |  |  |  |
| Quintile 1 (<4.2) | 316/3677 | 1.20 (1.01~1.41) | 0.036 |
| Quintile 2 (4.3–4.9) | 321/3513 | 1.16 (0.99~1.36) | 0.066 |
| Quintile 3 (5.0–5.7) | 434/4254 | 1.07 (0.93~1.23) | 0.358 |
| Quintile 4 (5.8–6.5) | 443/4123 | Ref. |  |
| Quintile 5 (>6.6) | 421/3780 | 1.27 (1.10~1.47) | <0.001 |
| P for trend | 1935/19437 |  | 0.460 |

**Notes:** The model was adjusted for age + sex+ race + education + BMI + poverty income ratio + smoking status + diabetes + hypertension + CVD + stroke+ medication usage + physical activity + alcohol use + HDL-C + TC + eGFR + healthy eating index

**Abbreviations:** BMI, body mass index; CVD, cardiovascular disease; TC, total cholesterol; HDL-C, high-density lipoprotein cholesterol; eGFR: estimated glomerular filtration rate

**eTable 3.** Hazard ratios (95% CIs) of all-mortality according to serum uric acid levels among hyperlipidemia patients with further adjustment of triglycerides and low-density lipoprotein cholesterol

| **Variable** | **Deaths/Total** | **Adjusted Model** | ***P-value*** |
| --- | --- | --- | --- |
|  | **n** | **HR (95% CI)** |  |
| Binary variable |  |  |  |
| Quintile 1 (<4.2) | 163/1702 | 1.52 (1.21~1.91) | <0.001 |
| Quintile 2 (4.3–4.9) | 174/1748 | 1.29 (1.04~1.59) | 0.021 |
| Quintile 3 (5.0–5.7) | 241/2147 | 1.15 (0.95~1.40) | 0.144 |
| Quintile 4 (5.8–6.5) | 193/1851 | Ref. |  |
| Quintile 5 (>6.6) | 230/1929 | 1.31 (1.08~1.60) | 0.007 |
| P for trend | 1001/9377 |  | 0.091 |

**Notes:** The model was adjusted for age + sex+ race + education + BMI + poverty income ratio + smoking status + diabetes + hypertension + CVD + stroke+ medication usage + physical activity + alcohol use + HDL-C + eGFR + healthy eating index + TG + LDL-C. TC was not included in the final model due to collinearity with TG and LDL-C.

**Abbreviations:** BMI, body mass index; CVD, cardiovascular disease; TC, total cholesterol; TG, triglyceride; HDL-C, high-density lipoprotein cholesterol; LDL-C, low-density lipoprotein cholesterol; eGFR: estimated glomerular filtration rate
